# Associations between lipid profiles and late‐life cognitive impairment among oldest‐old and centenarian adults

**DOI:** 10.1002/mco2.362

**Published:** 2023-09-07

**Authors:** Yujian Chen, Kaidi Yang, Ya Huang, Xuejiao Wang, Yali Zhao, Ping Ping, Shasha Guan, Shihui Fu

**Affiliations:** ^1^ Central Laboratory Hainan Hospital of Chinese People's Liberation Army General Hospital Sanya China; ^2^ Oncology Department Hainan Hospital of Chinese People's Liberation Army General Hospital Sanya China; ^3^ Blood Transfusion Department Hainan Hospital of Chinese People's Liberation Army General Hospital Sanya China; ^4^ Pediatric Department Hainan Hospital of Chinese People's Liberation Army General Hospital Sanya China; ^5^ General Station for Drug and Instrument Supervision and Control Joint Logistic Support Force of Chinese People's Liberation Army Beijing China; ^6^ Department of Cardiology Hainan Hospital of Chinese People's Liberation Army General Hospital Sanya China; ^7^ Department of Geriatric Cardiology Chinese People's Liberation Army General Hospital Beijing China

**Keywords:** centenarian, cognitive impairment, lipid profiles, oldest‐old

## Abstract

Dyslipidemia and cognitive impairment are common among old adults and the occurrence of them rises exponentially with increasing age. Evidences of the relationships between serum lipids and cognitive impairment are inconsistent or equivocal among older adults. This study aimed to investigate the associations between lipid profiles and late‐life cognitive impairment among oldest‐old and centenarian adults. In this cross‐sectional study, serum lipids were biochemically measured among 606 oldest‐old adults and 653 centenarians, and cognitive function was evaluated using mini‐mental state examination (MMSE). Multivariate linear and logistic regression analyses were performed to explore the associations between serum lipids and cognitive impairment. Results showed participants with cognitive impairment had lower total cholesterol (TC) levels compared with those without cognitive impairment (*p* < 0.05). TC levels were positively associated with MMSE (*p* < 0.05). Furthermore, a negative association was observed between TC levels and cognitive impairment (*p* for trend = 0.002). This negative association remained statistically significant after adjusting for confounders (*p* for trend = 0.028). These results suggested that older adults with higher TC levels were likely to have better cognitive function. Taking immoderate cholesterol‐lowering drugs among older adults is questionable and requires investigation, and cognitive performance of old adults with lower TC levels deserves more attention.

## Graphical abstract

Serum total cholesterol levels were negatively associated with later‐life cognitive impairment among oldest‐old and centenarian adults, suggesting that older adults with higher total cholesterol levels were likely to have better cognitive function. Taking immoderate cholesterol‐lowering drugs among older adults is questionable and requires investigation, and cognitive performance of old adults with lower total cholesterol levels deserves more attention.

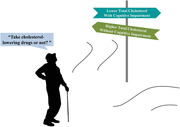

## 1 INTRODUCTION

Cognitive impairment, ranging from mild deficits to dementia, is a common manifestation of aging among older adults.^1^, ^2^ The occurrence of cognitive impairment rises exponentially with increasing age. Worldwide, mild cognitive impairment affects 10−15% of the population over the age of 65 years.^3^ With the rapid aging of the population, the number of older individuals living with cognitive impairment can be expected to rise.^4^ The incidence of dementia is 40.5% among older adults aged 90 years and above in China, whereas it is 1.5% in those aged 60−64 years.^5^ Cognitive impairment among older adults significantly affects their quality of life, contributes to disability‐related deaths, and receives extensive attention from researchers. Prevention strategies are particularly important to mitigating the increasing global burden of dementia, as the absence of a cure for the disease.^6^ Therefore, it is crucial to examine potentially modifiable risk factors for cognitive impairment among older population, in order to prioritize preventive approaches for cognitive impairment.

Lipid metabolism plays a significant role in neuronal development, synaptic plasticity and brain function.^7^, ^8^, ^9^ Serum lipids consist of several components including total cholesterol (TC), triglyceride (TG), high‐density lipoprotein cholesterol (HDL‐C), low‐density lipoprotein cholesterol (LDL‐C), apolipoprotein A (ApoA), and apolipoprotein B (ApoB). Cholesterol modulates the degradation process of the amyloid precursor protein in brain tissue, which plays a vital role in the development of dementia.^10^, ^11^ Cholesterol is a crucial component present in structures of dopamine transporters.^12^ Disruption of lipid homeostasis could therefore be an important factor in the progression of cognitive impairment and lipids may be sensitive biomarkers for early recognition of cognitive impairment.^4^, ^13^

Previous studies examined the relationships between serum lipids and cognitive impairment, but their results are inconsistent or equivocal among older adults. Some studies reported inverse associations between serum lipids and late‐life cognitive impairment, whereas other studies showed positive associations or no association. Pang et al.^14^ and Thorvaldsson et al.^15^ reported that lower TC levels were associated with relatively poorer cognition among individuals over 60 years. Ma et al.^16^ found that TC and LDL‐C were risk factors for cognitive function.^17^ van Exel et al.^4^ reported that HDL‐C levels were negatively associated with cognitive impairment among older population.^18^ However, Huang et al.^19^ reported that no significant relationships between serum lipids and cognitive function among older adults.^20^ Anstey et al.^21^ also claimed that TC and HDL‐C levels were not associated with cognitive impairment or any dementia. These conflicting results may vary due to variations in target populations including age, region, and ethnicity. Relationship between TC levels and the risk of dementia may differ depending on the age at measurement.^6^, ^21^, ^22^

In addition to those contradictory results above, evidences about the associations between serum lipids and cognitive function in individuals aged 80 years and above are still scarce. Therefore, a representative older cohort of adults aged 80 years or older may provide a more accurate determination of potential associations between serum lipids and cognitive impairment. This study based on the China Hainan Centenarian Cohort Study (CHCCS) aimed to investigate the associations between lipid profiles and late‐life cognitive impairment among oldest‐old and centenarian adults. Better knowledge of these associations between serum lipids and cognitive function may help decrease overall incidence of dementia in longevous people.

## 2 RESULTS

### 2.1 Baseline characteristics of participants

**FIGURE 1:**
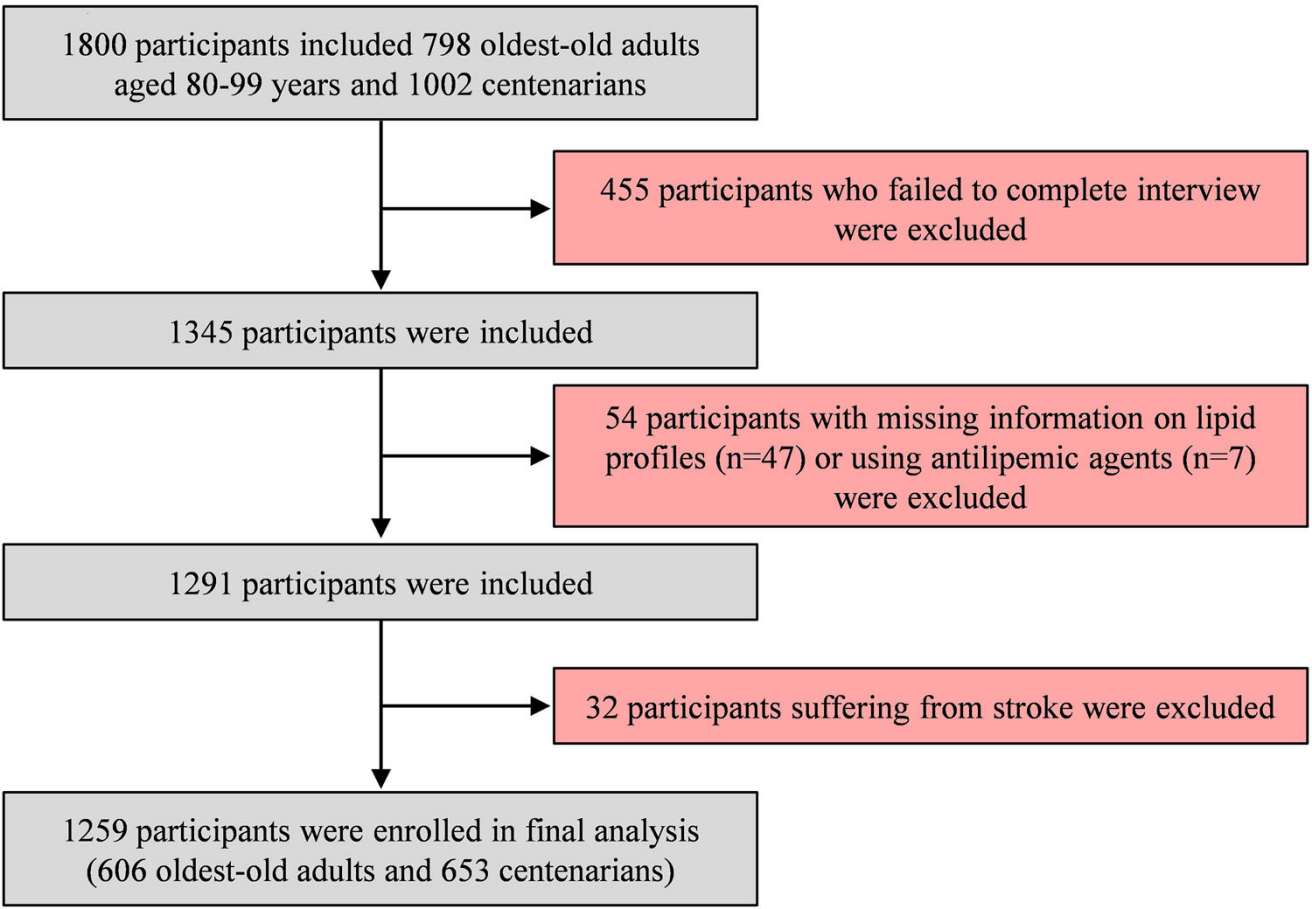
Flowchart of participant inclusion and exclusion.

A total of 1259 participants (606 oldest‐old adults and 653 centenarians) were included in this study (Figure 1). Table 1 illustrates that most of the participants were women (71.0%), lacked literacy (83.1%), and belonged to Han ethnicity (90.5%). They tended to live with their families (83.6%) and engage in social interaction (93.3%). One third of participants had activity of daily living (ADL) impairment and 68.4% had at least 1 h outdoor activity a day. The overall median of mini‐mental state examination (MMSE) was 11.0 (10.0), and the overall prevalence of cognitive impairment among the participants was 80.1%.

**TABLE 1:** Characteristics of all participants with and without cognitive impairment.

	All participants	Without cognitive impairment	With cognitive impairment	*p*
Number of participants	1259	250	1009	
*Demographic characteristics*				
Age, mean (s.d.) (years)	94.3 (9.5)	89.6 (9.0)	95.4 (9.3)	<0.001
Females (%)	71.0	52.4	75.6	<0.001
Illiteracy (%)	83.1	67.6	86.9	<0.001
Han ethnicity (%)	90.5	98.4	88.5	<0.001
*Lifestyle factors (%)*				
ADL impairment	33.1	8.0	39.3	<0.001
Outdoor activity > 1 h/d	68.4	89.8	63.1	<0.001
Living with families	83.6	78.4	84.9	0.013
Social interaction	93.3	96.4	92.5	0.030
Current cigarette smoking	7.6	12.4	6.4	0.002
Current alcohol drinking	13.5	17.8	12.5	0.032
Current tea drinking	16.2	31.9	12.3	<0.001
*Food consumption (%)*				
Meat	83.7	92.8	81.4	<0.001
Poultry	52.1	53.9	51.7	0.526
Fish	70.2	75.5	68.9	0.044
Milk	33.0	34.1	32.7	0.665
Egg	44.0	43.4	44.2	0.842
Fruit	72.3	67.8	73.4	0.083
Vegetable	97.3	99.6	96.7	0.012
*Physical examination, mean (s.d.)*				
SBP (mmHg)	150.4 (25.1)	150.3 (23.6)	150.4 (25.5)	0.982
DBP (mmHg)	77.3 (12.6)	79.8 (12.5)	76.7 (12.6)	<0.001
BMI (kg/m^2^)	19.6 (3.7)	20.6 (3.8)	19.3 (3.7)	<0.001
*Lipid profiles, median (interquartile range)*				
TC (mM)	4.75 (1.32)	5.00 (1.47)	4.71 (1.28)	0.003
TG (mM)	1.04 (0.65)	1.12 (0.69)	1.03 (0.63)	0.136
HDL‐C (mM)	1.40 (0.53)	1.42 (0.58)	1.39 (0.52)	0.596
LDL‐C (mM)	2.83 (1.11)	3.05 (1.10)	2.78 (1.10)	0.001
ApoA (g/L)	1.40 (0.38)	1.44 (0.36)	1.40 (0.39)	0.054
ApoB (g/L)	0.98 (0.37)	1.06 (0.38)	0.96 (0.36)	<0.001
*Cognitive function, median (interquartile range)*				
MMSE	11.0 (10.0)	24.0 (5.5)	9.0 (7.0)	<0.001

Abbreviations: ADL, activity of daily living; ApoA, apolipoprotein A; ApoB, apolipoprotein B; BMI, body mass index; DBP, diastolic blood pressure; HDL‐C, high‐density lipoprotein cholesterol; LDL‐C, low‐density lipoprotein cholesterol; MMSE, mini‐mental state examination; SBP, systolic blood pressure; TC, total cholesterol; TG, triglyceride.

### 2.2 Characteristics of participants with and without cognitive impairment

**FIGURE 2:**
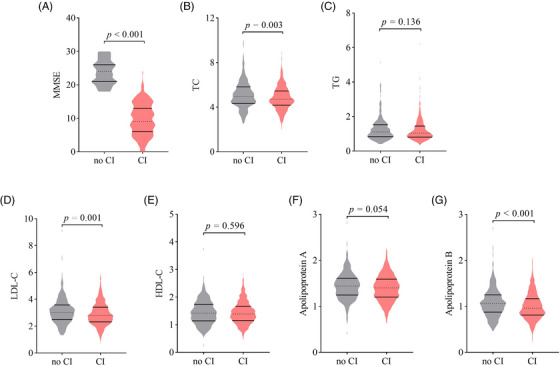
Relationships between lipid profiles and late‐life cognitive impairment (CI) among Chinese oldest‐old and centenarian adults. (A) Mini‐mental state examination (MMSE) was lower in participants with CI than in those without CI (no CI); (B) total cholesterol (TC) levels were lower in participants with CI than in those without CI (no CI); (C) triglyceride (TG) levels had no difference between participants with CI and those without CI (no CI); (D) low‐density lipoprotein cholesterol (LDL‐C) levels were lower in participants with CI than those without CI (no CI); (E) high‐density lipoprotein cholesterol (HDL‐C) levels had no difference between participants with CI and those without CI (no CI); (F) apolipoprotein A levels had no difference between participants with CI and those without CI (no CI); (G) apolipoprotein B levels were lower in participants with CI than in those without CI (no CI).

Table 1 compares the characteristics of participants with and without cognitive impairment. The older (95.4 vs. 89.6 years), females (75.6 vs. 52.4%), illiteracy (86.9 vs. 67.6), and non‐Han ethnicity (11.5 vs. 1.6%) were more likely to have cognitive impairment (*p* < 0.001 for all). Compared with those without cognitive impairment, participants with cognitive impairment had a higher prevalence of ADL impairment (*p* < 0.001) and were more likely to live with families (*p* = 0.013), but were less likely to participate in outdoor activity *(p* < 0.001) and maintain social interaction (*p* = 0.030). Regular consumption of meat, fish, and vegetable was less common in participants with cognitive impairment than in those without cognitive impairment (*p*s < 0.05). Diastolic blood pressure (DBP), body mass index (BMI), TC, LDL‐C, and ApoB levels were lower in participants with cognitive impairment than in those without cognitive impairment (*p*s < 0.05). Difference in MMSE and serum lipids of participants with and without cognitive impairment was shown in Figure 2.

### 2.3 Associations between lipid profiles and cognitive impairment

As Table 2 shows, TC levels were positively associated with MMSE in multivariate linear regression analyses with and without adjustment for confounders (*p*s < 0.05). As Table 3 shows, a negative association was observed between TC levels and cognitive impairment in multivariate logistic regression analyses (Model 1, *p* for trend = 0.002). This negative association remained significant after adjusting for confounders (Model 2, *p* for trend = 0.028). A negative association was also found between ApoB levels and cognitive impairment (Model 1, *p* for trend = 0.004), but the association was not significant after adjusting for confounders (*p* for trend = 0.791).

**TABLE 2:** Significant associations with MMSE in multivariate linear regression analyses.

	Beta	95%CI		*P*
TC				
Model 1	0.878	0.693	1.063	<0.001
Model 2	0.457	0.274	0.640	0.013

Abbreviations: CI, confidence interval; MMSE, mini‐mental state examination; TC, total cholesterol.

Model 1 was not adjusted.

Model 2 was adjusted for demographic characteristics, lifestyle factors, food consumption, physical examination, and blood analyses listed in Table 1.

**TABLE 3:** ORs for cognitive impairment over the strata of lipid levels in multivariate logistic regression analyses.

	Lower	Middle	Higher	*p* for trend
TC				
Range	<5.2 mmol/L	5.2–6.2 mmol/L	≥6.2 mmol/L	
Model 1	1.000 (Reference)	0.778 (0.560, 1.080)	0.538 (0.358, 0.809)	0.002
Model 2	1.000 (Reference)	0.580 (0.322, 1.043)	0.358 (0.124, 1.032)	0.028
TG				
Range	<1.70 mmol/L	1.70–2.3 mmol/L	≥2.3 mmol/L	
Model 1	1.000 (Reference)	1.261 (0.779, 2.070)	0.678 (0.392, 1.171)	0.332
Model 2	1.000 (Reference)	1.611 (0.836, 3.105)	0.885 (0.416, 1.883)	0.766
HDL‐C				
Range	<1.0 mmol/L	–	≥1.0 mmol/L	
Model 1	1.000 (Reference)	–	1.206 (0.812, 1.792)	0.353
Model 2	1.000 (Reference)	–	1.310 (0.663, 2.590)	0.348
LDL‐C				
Range	<3.4 mmol/L	3.4–4.1 mmol/L	≥4.1 mmol/L	
Model 1	1.000 (Reference)	0.924 (0.640, 1.333)	0.648 (0.416, 1.009)	0.076
Model 2	1.000 (Reference)	1.524 (0.772, 3.006)	1.801 (0.620, 5.237)	0.105
Apo A				
Range	<1.2 g/L	1.2–1.6 g/L	≥1.6 g/L	
Model 1	1.000 (Reference)	0.787 (0.550, 1.125)	0.778 (0.516, 1.173)	0.243
Model 2	1.000 (Reference)	1.065 (0.616, 1.841)	1.007 (0.525, 1.930)	0.916
Apo B				
Range	<0.8 g/L	0.8–1.1 g/L	≥1.1 g/L	
Model 1	1.000 (Reference)	0.852 (0.575, 1.261)	0.588 (0.396, 0.875)	0.004
Model 2	1.000 (Reference)	0.940 (0.555, 1.591)	1.056 (0.502, 2.222)	0.791

Abbreviations: ApoA, apolipoprotein A; ApoB, apolipoprotein B; HDL‐C, high‐density lipoprotein cholesterol; LDL‐C, low‐density lipoprotein cholesterol; ORs, odds ratios; TC, total cholesterol; TG, triglyceride.

Model 1 and model 2 were shown with OR (95%confidence interval).

Model 1 was not adjusted.

Model 2 was adjusted for demographic characteristics, lifestyle factors, food consumption, physical examination and blood analyses listed in Table 1.

## 3 DISCUSSION

In this study, the associations between lipid profiles and cognitive impairment were explored among Chinese oldest‐old and centenarian adults. An inverse association was observed between TC levels and cognitive impairment, and this association remained significant after adjusting for potential confounders. Those results expanded our knowledge that higher TC levels are associated with better late‐life cognitive function among older adults. Multivariate statistical analyses showed that TC rather than other serum lipids was an indicator of cognitive impairment, suggesting that TC is a more significant indicator of cognitive impairment than other serum lipids. Different serum lipids are interrelated factors between each other, and TC is their better representative to indicate cognitive impairment.

To date, evidences for the association between TC levels and cognitive function were conflicting. Some studies reported that TC levels were negatively, positively, or not associated with cognitive impairment.^14^, ^15^, ^16^, ^19^, ^20^, ^23^, ^24^ Our results on the association between TC and cognition were consistent with several studies. A more recent study suggested that higher TC levels were statistically associated with improved cognitive function among females over 60 years old.^14^ Research based on a representative sample of participants aged 70 years and older revealed that both lower TC levels and decreased TC levels were related to relatively poorer cognition.^15^ In an 18‐year longitudinal study of individuals aged 70 years and older, a significant association was observed between higher TC levels and a decreased dementia.^25^ Another study in Shanghai also reported that TC levels were inversely associated with incident dementia among older adults aged ≥60 years.^24^ Some hypotheses may explain the observed relationship between TC and cognition among older adults. First, higher TC levels in late‐life may be an indicator of better health status. People survive to old age with higher TC levels may be more robust and therefore comparatively invulnerable to cognitive impairment.^24^, ^25^, ^26^, ^27^ Meanwhile, cholesterol is a precursor of steroid hormones, provides integrity of cell structure and modulates fluidity of cell membranes, which is essential for synaptic integrity and neural transmission.^28^, ^29^, ^30^, ^31^ These processes are compromised with aging development and decreased cholesterol may expedite their dysfunction. Further testing of these hypotheses is warranted through appropriately designed, randomized, and controlled trials.

By contrast, some studies revealed harmful effects of cholesterol on cognition. Anstey et al.^21^ found that higher mid‐life TC levels increases the risk of late‐life dementia and correlates with the onset of dementia. A cross‐sectional study including 1754 Chinese adults aged 55−80 years showed that higher TC levels were associated with poorer cognitive function, especially in aging female participants.^32^ Another research including participants aged 54−91 years also reported the same association: people with mild cognitive impairment had higher TC levels than the control group.^23^ Ma et al.^16^ reported that higher TC and LDL‐C levels were markedly associated with greater cognitive impairment in Chinese adults aged over 60 years. In addition, some studies suggested no relationship between TC levels and cognitive impairment among older adults.^20^, ^26^ The contradictory findings could potentially be attributed to the timing of cholesterol measurements relative to age. Furthermore, different region and ethnicity may partly explain the discrepancy in these findings. Several studies revealed that higher TC levels measured in mid‐life, but not late‐life, is a risk factor for cognitive impairment.^6^, ^21^, ^25^, ^33^ Age is a significant risk determinant of cognitive impairment,^5^, ^34^ and cognitive impairment continues to rise exponentially after the age of 90 years.^35^ Our study showed that higher TC levels were inversely associated with cognitive impairment among Chinese oldest‐old and centenarian adults. On the evidence of our data, cholesterol may be a significantly protective factor for late‐life cognitive impairment. Given the growing interest in controlling cholesterol among older adults, it is crucial to explore underlying mechanism of this association. If cholesterol indeed plays a protective role against cognitive impairment among oldest‐old and centenarian adults, it may be necessary to reevaluate the risk‐benefit ratio of lowering cholesterol in this population. Besides, changes in serum TC levels may potentially serve as an indicator for cognitive impairment among older adults, but further research is needed to confirm this.

There are some limitations of this study that need to be acknowledged. First, a lack of causal relationship between TC levels and cognitive function may exist due to the cross‐sectional study design. Second, MMSE was used to define cognitive impairment. MMSE is not considered an optimal diagnostic tool for cognitive impairment. Instead, a comprehensive neuropsychological assessment would be preferable. Third, this study only included Chinese participants, so it could not be generalized to other ethnicities. Finally, this study studied oldest‐old and centenarian adults, among whom 83.1% were illiterate. Age and education were significant factors affecting cognitive impairment. Hence, a more general and comprehensive older cohort should be performed with follow‐up to verify and supplement the findings. In the upcoming research, we will include a greater number of older individuals with more broader age groups and varying educational backgrounds for further validation.

## 4 CONCLUSIONS

In summary, our study found a negative association between TC levels and cognitive impairment among Chinese oldest‐old and centenarian adults, suggesting that older adults with higher TC levels were likely to have better cognitive function. The results indicated that taking immoderate cholesterol‐lowering drugs among older adults is questionable and requires investigation, and cognitive performance of old adults with lower TC levels deserves more attention.

## 5 METHODS

### 5.1 Study design

The participants for this study were recruited from a community‐based study of the CHCCS with all inhabitants aged ≥80 years residing in Hainan Province, China, from June 2014 to December 2016.^36^ Details of this study including survey design, interview procedures and sampling strategy have been described previously.^36^, ^37^ To permit valid comparisons with the Chinese Longitudinal Healthy Longevity Survey, the China Health and Retirement Longitudinal Study, and other national and international epidemiological studies of older populations, the CHCCS deliberately used standardized and validated instruments for data collection. Standard procedures were followed during the home interview, physical examination, and blood analyses. In brief, 1800 participants included 798 oldest‐old adults aged 80−99 years and 1002 centenarians (Figure 1). There were 455 participants who failed to complete interview because of physical weakness, hearing disorder, speech impediment, poor eyesight, or other personal reasons excluded from data analyses. There were 54 participants who were excluded because of missing information on lipid profiles (47 participants) or using antilipemic agents (seven participants). There were 32 individuals suffering from stroke also excluded, and 1259 participants (606 oldest‐old adults and 653 centenarians) were enrolled in the final analyses. The study protocol was approved by the Ethics Committee of Hainan Hospital of Chinese People's Liberation Army General Hospital (Sanya, Hainan; Number: 301HNLL‐2016‐01). All participants provided written informed consent. For cognitively impaired participants, informed consent was obtained from their guardians.

### 5.2 Cognitive function

Cognitive function was evaluated using MMSE,^38^, ^39^, ^40^ which is a well‐known and widely recognized method for quantitatively assessing cognitive impairments. Education is a significant demographic factor influencing MMSE.^41^ The optimal cut‐off points for defining cognitive impairment were determined based on education levels and established based on previous literatures.^42^, ^43^, ^44^ In these literatures, MMSE was proved to be feasible among Chinese for cognitive impairment screening. Cognitive impairment was defined as: MMSE ≤ 17 for illiteracy; MMSE ≤ 20 for primary school (1–6 years of education), and MMSE ≤ 24 for middle or higher school (more than 6 years of education).^42^, ^43^, ^44^

### 5.3 Serum lipids

Venous blood samples collected from each participant were transported in a cold storage (4°) to the Clinical Laboratory and assayed within 6 h. Serum TC, TG, HDL‐C, and LDL‐C levels were analyzed on a fully automated biochemical analyzer (Roche Cobas c702) using a standard procedure. Serum lipids were categorized into two or three strata based on the criteria provided by the Chinese Medical Association 2016 (Table 4).

**TABLE 4:** Strata of serum lipids based on the criteria provided by the Chinese Medical Association 2016.

	Lower	Middle	Higher
TC (mM)	<5.2	5.2–6.2	≥6.2
TG (mM)	<1.70	1.70–2.3	≥2.3
HDL‐C (mM)	<1.0	–	≥1.0
LDL‐C (mM)	<3.4	3.4–4.1	≥4.1
ApoA (g/L)	<1.2	1.2–1.6	≥1.6
ApoB (g/L)	<0.8	0.8–1.1	≥1.1

Abbreviations: ApoA, apolipoprotein A; ApoB, apolipoprotein B; HDL‐C, high‐density lipoprotein cholesterol; LDL‐C, low‐density lipoprotein cholesterol; TC, total cholesterol; TG, triglyceride.

### 5.4 Possible confounders

Potential confounders were selected based on previous studies^19^, ^45^, ^46^, ^47^ and obtained from structured questionnaire and standard survey. The confounders included demographic characteristics, lifestyle factors, food consumption, physical examination, and blood analyses, which may be associated with or play effects on both lipid profiles and cognitive impairment (Table 1). Therefore, potential confounders were controlled when the associations were assessed between serum lipids and cognitive impairment. Education levels were dichotomized into illiteracy versus primary school or above. Ethnicity was dichotomized into Han ethnicity (the predominant ethnicity in China) versus non‐Han ethnicity. Cigarette smoking, alcohol drinking, and tea drinking were categorized as current, former or never. ADL was scored based on Barthel Index,^48^ and ADL impairment was defined as a score of less than 90. Outdoor activity was described as engaging in daily outdoor walking or gardening for more than 1 h. Living condition referred to whether an individual resided with their family or not. Social interaction was defined as meeting and communicating with at least one relative or friend once a month. Regular consumption of food was defined as consumption of the food at least once per week. Systolic blood pressure (SBP) and DBP were respectively measured twice by trained physicians, and mean value of two measurements was used to calculate the final levels of blood pressure. BMI was calculated by dividing body weight in kilograms by the square of height in meters.

### 5.5 Statistical analyses

Continuous variables with normal distribution were described as mean and standard deviation (s.d.) and compared using the Student's *t*‐test. Continuous variables with skewed distribution were described as median and interquartile range and compared using the Mann–Whitney *U* test. Categorical variables were described as number and percentage and compared with Chi‐square test. Multivariate linear regression analyses (stepwise) were employed to assess the relationships between serum lipids and MMSE (continuous variables). Multivariate logistic regression analyses (Enter) were employed to evaluate the relationships between serum lipids and cognitive impairment (categorical variables). Model 1 remained unadjusted, whereas Model 2 was adjusted for demographic characteristics, lifestyle factors, food consumption, physical examination, and blood analyses listed in Table 1. Odds ratios (ORs) > 1.00 indicated increased odds of cognitive impairment, whereas ORs < 1.00 indicated lower odds of cognitive impairment. *P* values for trend were calculated by including the ordinal lipid variables as categorical variables (the strata of lipid levels) in multivariate logistic regression analyses. All statistical analyses were performed with the Statistic Package for Social Science (SPSS) version 22.0 (SPSS Inc.). Two‐sided *p* values less than 0.05 were considered statistically significant.

## AUTHOR CONTRIBUTION

Y. C. conceived and designed the project, draft and revised the paper. Y. C., K. Y., Y. H., X. W., and S. G. collected the data and conducted the data analyses. Y. Z., P. P., S. G., and S. F. supervised the analyses and suggested revisions of the paper. All the authors have read and approved the final manuscript.

## CONFLICT OF INTEREST STATEMENT

The authors declare no conflicts of interest.

## ETHICS STATEMENT

The ethics committee of Hainan Hospital of Chinese People's Liberation Army General Hospital (Sanya, Hainan; No.301HNLL‐2016‐01) approved this study protocol. Written informed consent was obtained from all individuals who participated in this study. For cognitively impaired participants, informed consent was obtained from their guardians.

## Data Availability

The data supporting this study are available from the corresponding author for reasonable request.
